# ADAM17 promotes the invasion of hepatocellular carcinoma via upregulation MMP21

**DOI:** 10.1186/s12935-020-01556-6

**Published:** 2020-10-21

**Authors:** Yuqi Xiang, Liyu Liu, Ying Wang, Bo Li, Jinwu Peng, Deyun Feng

**Affiliations:** 1 Department of Pathology, Xiangya Hospital, Central South University, Changsha, 410008 Hunan China; 2 Department of Pathology, Xiangya Basic Medical School, Central South University, Changsha, 410008 Hunan China; 3 Key Laboratory for Molecular Radiation Oncology of Hunan Province, Center for Molecular Medicine, Xiangya Hospital, Central South University, Changsha, 410008 Hunan China; 4 Department of Pathology, Xiangya Changde Hospital, Changde, 415000 Hunan China

**Keywords:** HCC, ADAM17, MMP21, Metastasis

## Abstract

**Background:**

The upregulation of ADAM17 has been reported to be associated with invasion and metastasis in various tumors, however the molecular mechanism of ADAM17 in the progression of hepatocellular carcinoma (HCC) remain to be clarified. Human matrix metalloproteinase 21 (MMP21), the newest member of the MMP gene family, has been suggested to play an important role in embryogenesis and tumor progression. So far, nothing is known about the relationship between ADAM17 and MMP21.

**Methods:**

In this study, the expression level of ADAM17 and MMP21 in HCC tissues was measured by immunohistochemistry. The Scratch wounding assay and Transwell were used to identify the invasion and metastasis ability. ELISA was used to evaluate the production of MMP21. Coimmunoprecipitation experiments demonstrated a direct association between ADAM17 and MMP21. HPLC was used to confirmed that ADAM17 participated in the maturation of MMP21.

**Results:**

Our present data indicated that ADAM17 and MMP21 was significantly upregulated in human HCC tissues. Knockdown of ADAM17 in HCC inhibited cell invasion and metastasis. Moreover, ADAM17 regulates the secretion and expression of MMP21. Furthermore we discovered a direct association between ADAM17 and MMP21, and we also found MMP21 prodomain could be cleaved by ADAM17.

**Conclusion:**

Our data suggest that ADAM17 plays an important role in the development of HCC invasion and metastasis and this function may be implement by MMP21.

## Background

Hepatocellular carcinoma (HCC) is a highly aggressive and heterogeneous disease. The latest national cancer statistics released by the National Cancer (China) showed that the morbidity of hepatocellular carcinoma is 36.5 per 10^4^ people and that it ranks fourth in malignant tumors [1, 2]. Therefore, we explored the mechanisms underlying the carcinogenesis and progression of HCC to benefit therapy.

ADAM17 was initially identified as an important member of the ADAM family by Black RA’s team in 1997. Because the enzyme is responsible for releasing soluble tumor necrosis factor-alpha (TNFα) from the plasmalemma, it is also known as TNFα converting enzyme (TACE/ADAM17) [3]. It has been reported that the dysregulation of ADAM17 contributes to the pathology of various cancers. For instance, ADAM17 protein was highly expressed in esophageal squamous cell carcinoma (ESCC) and promoted the development, invasion and metastasis of ESCC [4–7]. Similarly, ADAM17 silencing suppressed the invasion and proliferation of MCF7 cells in vitro [8]. Furthermore, Wang H P’s study suggested that Nox1 promoted colorectal cancer metastasis by stabilizing ADAM17 [9]. Although several studies have shown that ADAM17 promotes the occurrence and development of hepatocellular carcinoma [10, 11], the potential regulatory mechanism has not been fully elucidated.

MMP21 is the last uncharacterized MMP which is different from other MMPs and is rarely produced in normal tissues without inflammatory or oncogenic stimuli, which may implicate a role for MMP21 in normal tissue homeostasis [10]. The prodomain of MMP21 contains a peptide sequence similar to that of TNFα. It has also been reported that MMP21 is expressed in cancer cells located in the invasive front of tumors rather than dysplastic cells and enhances tumor metastasis in some solid tumors [12–15]. Positive correlations between MMP21 and tumor diameter, depth of invasion, vessel invasion,lymph node distant metastases,and tumor-node-metastasis stage were observed in gastric cancer. The overall survival rate was significantly lower in MMP21- and MMP28- positive patients [15]. However, the expression pattern of MMP21 in HCC and whether MMP21 could be activated by other proteinases remain unknown. Notably, in this study, we investigated whether ADAM17 may regulate the maturation of MMP21 and influence the progression of HCC.

## Methods

### Patients and specimens

All experimental procedures involving the use of human tissue included the relevant receipt of written informed consent and were approved by the institutional review board at The Xiangya Hospital of Centre South University. For formalin-fixed paraffin-embedded HCC samples, human specimens were collected from the XiangYa Hospital tissue biobank, and the protocol for staining was approved by the local ethics committee of The Xiangya Hospital.

### Immunohistochemistry and evaluation of immunostaining intensity

The tumor tissue was subjected to immunohistochemistry and stained with primary antibodies against ADAM17 (Abcam, ab2051, 1:100) and MMP21 (Abcam, MAB2079Z, 1:200) followed by light microscopy examination. The immunostaining intensity for the two proteins was reviewed and independently scored by pathologists who were blinded to the clinical data and scored independently according to the staining intensity and the proportion of stained tumor cells. According to the staining intensity, samples were scored as follows: no staining = 0; light yellow (weak staining) = 1; yellow brown (moderate staining) = 2; and brown (strong staining) = 3. The scores were expressed in terms of the proportion of cell staining as follows: scores of 0, 1, 2, and 3 indicated 0, ≤ 30%, 30%–70% and ≥ 70% positive cells, respectively. Thus, the two combined scores (from the two independent pathologists) were taken as the final score, where 0 indicated negative (−); 1–2, weak positive (+); 3–4, strong positive (++); and 5–6, very strong positive (+++). In the statistical analyses, (++) and (+++) were classified as the positive group, while (−) and (+) were classified as the negative group.

### Cell culture

Human HCC MHCC97H and Huh7 cells were cultured in DMEM supplemented with 10% fetal calf serum and 1% penicillin/streptomycin at 37 °C in a humidified atmosphere of 5% CO_2_. Human HCC SMMC7721 cells were maintained in RPMI-1640 medium supplemented with 10% fetal bovine serum and 1% penicillin/streptomycin.

### Tranfection assay

Specific small interfering RNA (siRNA) against ADAM17 (si-ADAM17) and MMP21 (siMMP21) and siRNA scrambled control (si-NC), were purchased from RIBBIO (Shanghai, China). Huh7 and SMMC7721 cells were transfected with plasmids or oligonucleotides using DharmaFECT Reagent (Invitrogen, Carlsbad, CA, USA). The sequences for siADAM17, siMMP21,siNC were as follows: siADAM17: 5′-GCTTGTTCATCGAGTGAAA-3′, 5′-GGATGGTCTAGCAGAATGT-3′; siMMP21: 5′-GATCCATAATGCAACCAAA-3′, 5′-ACTGGAAGGTAGTTAATGA-3′; siNC: TTCTCCGAACGTGTCACGTTT.

### Cell invasion assay

For the transwell invasion assay, 24-well transwell units with an 8-μm pore size polycarbonate filter (Millipore) were used according to the manufacturer’s instructions. Briefly, filters were coated with Matrigel to form a continuous thin layer. Then, cells were seeded in DMEM in the upper chamber. The lower chamber was filled with DMEM with 10% FBS. Following 24 h of incubation at 37 °C, cells remaining in the upper compartment were removed using cotton swabs. The cells that invaded through the filter into the lower compartment were fixed with 4% paraformaldehyde and stained with crystal violet (0.5% in 20% methanol). To quantify invasive cells, three independent fields of invasive cells per well were photographed.

### Scratch wounding assay

Transfected cells were plated in six-well plates and incubated at 37 °C until a confluent monolayer was formed (> 90%). With a 100-µl sterile pipette tip, a scratch was created. The cells were washed three times with PBS (pH 7.2) to remove cell fragments, and low serum DMEM was added. Micrographs were taken immediately after wounding and after 24 h, 48 h, 72 h, and 110 h. The closure percentage was calculated using the following equation: closure percentage = [1–(Tx/T0)] × 100%, where T0 is the wounded area at 0 h and Tx is the wounded area after x h.

### Western blot

Cells were then harvested in lysis buffer. A BCA protein assay kit was used to determine the concentration of protein. Samples were separated on a 10% SDS–PAGE gel, followed by transfer to polyvinylidene difluoride (PVDF) membranes in an electrophoretic manner. The primary antibodies were used at a 1:1000 dilution, the loading control anti-tubulin was used at a 1:5000 dilution, and the secondary antibody was used at a 1:1000 dilution. The targeted proteins in the membrane were detected with an electrochemiluminescence detection system followed by exposure to X-ray film.

### Elisa

To evaluate the production of MMP21 substrates, the transfected cells were prepared. ELISA kits (CUSA-BIO, Catalog Number. CSB-EL014668HU) were used to analyze the collected medium specimens for the proteins of interest. Supernatants were collected in triplicate for each cell line.

### Immunoprecipitation

Cells underwent cytolysis in 1 ml of RIPA buffer, followed by a 10-min incubation on ice. Total cell lysates were centrifuged for 10 min at 20,000*g* at 4 °C. Ten microliters of primary antibody was used to incubate the supernatants for 60 min. Then, 20 µl of protein A/G PLUS-agarose was added to the lysate and incubated at 4 °C overnight. The beads were washed with ice-cold RIPA buffer four times. The samples were then suspended and denatured in SDS sample buffer (which contained 100 mM dithiothreitol, 10% glycerol, 50 mM Tris, pH 6.8, 2% SDS, and 0.01% bromophenol blue).

### HPLC method

Recombinant human TACE/ADAM17 (rhTACE) (Catalog # 930-ADB) was purchased from R&D Systems. The amino acid polypeptide sequence (ALAQAVRRFQ) was ordered from Sangon Biotech. The assay buffer used in this study was 25 mM Tris, 2.5 µM ZnCl2, 0.005% Brij-35 (w/v), pH 9.0. First, rhTACE was diluted to 0.2 ng/µl in assay buffer. Second, the substrate was diluted to 20 µM in assay buffer. Third, 50 µl of 0.2 ng/µL rhTACE was added, and the reaction was initiated by adding 50 µl of 20 µM substrate. A substrate blank was included that contained 50 μl of assay buffer and 50 µl of substrate. The HPLC reaction conditions in our study were as follows: column: 250*4.6 mm, Sinchrom ODS-BP-5; solvent A: 0.1% TFA in 100% water; solvent B: 0.1% TFA in 100% acetonitrile; flow rate: 1.0 ml/min; wavelength (nm): 220; and volume: 10 µl.

### Statistical analysis

SPSS 21.0 software was used for statistical analysis. Chi-square test or Fisher Exact test are used to compare the expressions of ADAM17 and MMP21 in cancer tissues and adjacent tissues. The relationship between the two indexes and the clinical data of hepatocellular carcinoma patients was analyzed by chi-square test. Spearman rank correlation analysis was used to analyze the expression correlation between ADAM17 and MMP21. P < 0.05 was considered statistically significant. Each experiment was repeated three times.

## Results

### ADAM17 and MMP21 are significantly upregulated in human HCC tissues, and there is a positive correlation between the two genes

**Fig. 1:**
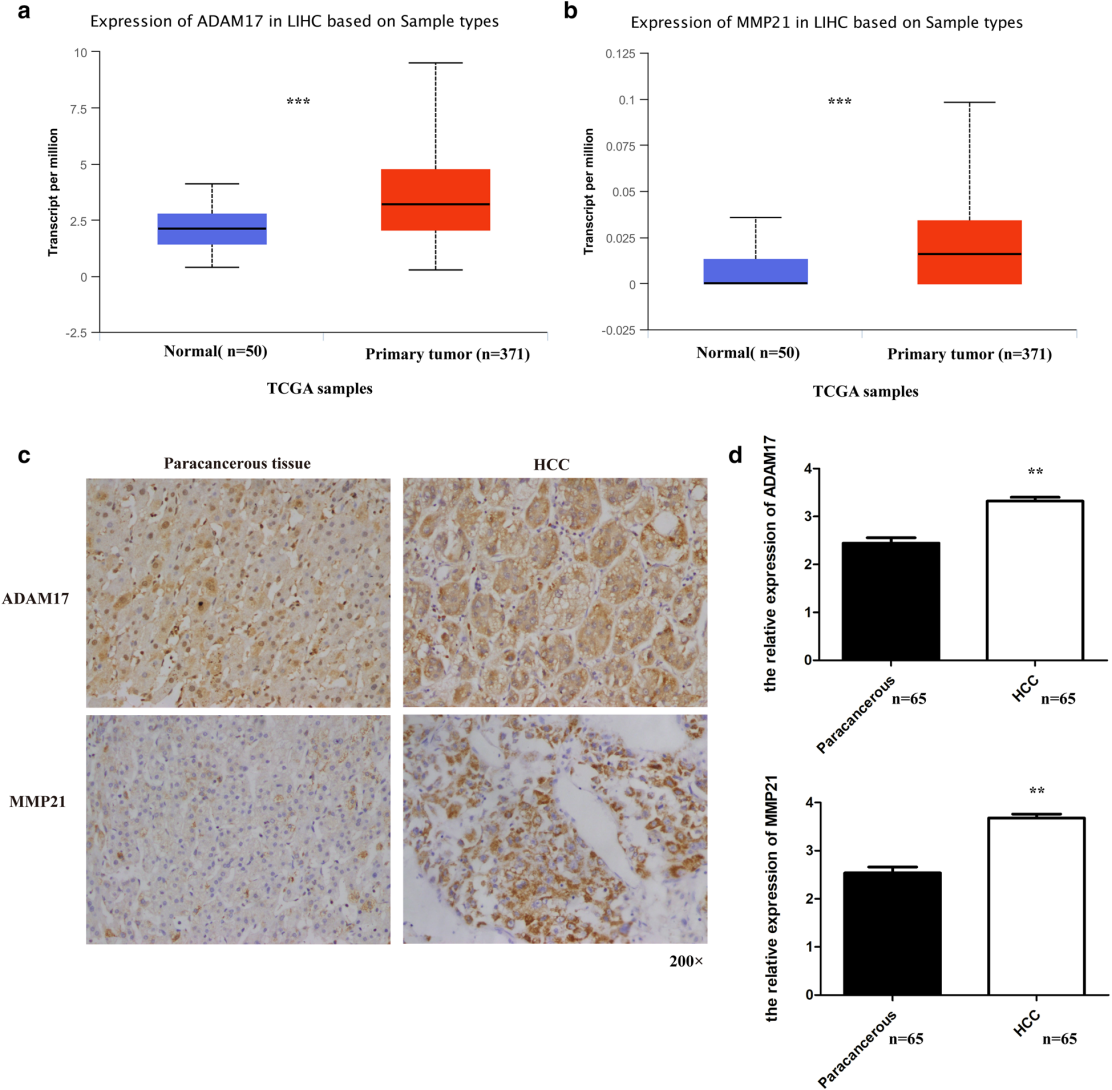
Levels of ADAM17 and MMP21 in patients with HCC. **a** High expression of ADAM17 in tumor tissue of patients with HCC compared with normal tissue in TCGA samples. **b** The high expression of MMP21 in tumor tissue of patients with HCC compared with normal tissue in TCGA samples. **c** Representative micrographs of immunohistochemical staining of ADAM17 and MMP21 in HCC tissues and adjacent liver tissue are shown. **d** Expression levels of ADAM17 and MMP21 in HCC tissues (n = 65) and adjacent liver tissue (n = 65) (*t*-test). (***P < 0.001, **P < 0.01, *P < 0.05)

To examined the expression of ADAM17 and MMP21 in patients with HCC. First, we analyzed the UALCAN database and found that the expression level of ADAM17 in primary tumors was higher than that in normal tissues (Fig. 1a). Subsequently, we analyzed the expression of MMP21, and the result was consistent with ADAM17 (Fig. 1b). To further confirm this discovery, we performed immunohistochemistry to investigate the expression of ADAM17 and MMP21 in human HCC tissues and adjacent tissues (Fig. 1c). As shown in Fig. 1c, d, the expression levels of ADAM17 and MMP21 were both higher in HCC tissues than in adjacent liver tissues (P < 0.001) (Table 1). Our statistical analysis showed that there was a positive correlation between ADAM17 and MMP21 in HCC (Table 2). Furthermore, we evaluated the correlation of ADAM17 and MMP21 with the patients’ clinicopathological parameters and found that the expression of MMP21 was related to the patient’s microvascular invasion, and the results were statistically significant (Table 3). Although there was no significant correlation between ADAM17 and other clinicopathological parameters in HCC, the expression of ADAM17 may be correlated with microvascular invasion, as the *P* value is 0.063. In conclusion, our results suggest that ADAM17 and MMP21 may play an important role in the development of HCC.

**Table 1:** Expression of ADAM17 and MMP21 in hepatocellular carcinoma and paracancerous tissues(Chi-square test)

Tissues	ADAM17 expression		High expression rate (%)	P
	Low expression	High expression		
A				
Paracancerous tissues	28	37	56.92	<0.001
HCC tissues	5	60	92.31	
Tissues	MMP21 expression		High expression rate (%)	P
	Low expression	High expression		
B				
Paracancerous tissues	34	31	47.69	<0.001
HCC tissues	7	58	89.23

**Table 2:** The correlation of ADAM17 and MMP21 expression in hepatocellular carcinoma (Spearman test)

MMP21 expression	ADAM17 expression					
	(−)	(+)	(++)	(+++)	P	r
High expression	2	3	35	25	0.039	0.2201
Low expression	1	6	8	50

**Table 3:** Relationship between the level of ADAM17 and active MMP21 and variable Clinicpathological features (Chi-square test)

Tissues	n	ADAM17 expression		High expression rate (%)	P
		Low expression	High expression		
Gender					
Male	54	4	50	92.59	0.849
Female	11	1	10	90.90	
Age (year)					
> 50	30	1	29	96.67	0.222
≤ 50	35	4	31	88.57	
Tumor number					
Single	52	5	47	90.38	0.574
Multiple	13	0	13	100	
Tumor size (cm)					
≤ 3	12	0	12	100	0.575
> 3	53	5	48	90.57	
Histology					
Well	4	1	3	75	0.346
Moderately	43	2	41	95.35	
Moderately-Poor	13	1	12	92.31	
Poor	5	1	4	80	
Microvascular invasion					
Yes	38	1	37	97.37	0.069
No	27	4	23	85.19	

Tissues	n	MMP21 expression		High expression rate (%)	P
		Low expression	High expression		
Gender					
Male	54	5	49	90.74	0.384
Female	1	2	9	81.82	
Age(year)					
> 50	30	3	27	90	0.853
≤ 50	35	4	31	88.57	
Tumor number					
Single	52	5	47	90.38	0.548
Multiple	13	2	11	84.62	
Tumor size (cm)					
≤ 3	12	2	10	83.33	0.604
> 3	53	5	48	90.57	
Histology					
Well	4	1	3	75	0.171
Moderately	43	2	41	95.35	
Moderately- Poor	13	3	10	76.92	
Poor	5	1	4	80	
Microvascular invasion					
Yes	41	1	40	97.56	0.005
No	24	6	18	75	

### ADAM17 promotes the viability and migration of hepatocellular carcinoma

**Fig. 2:**
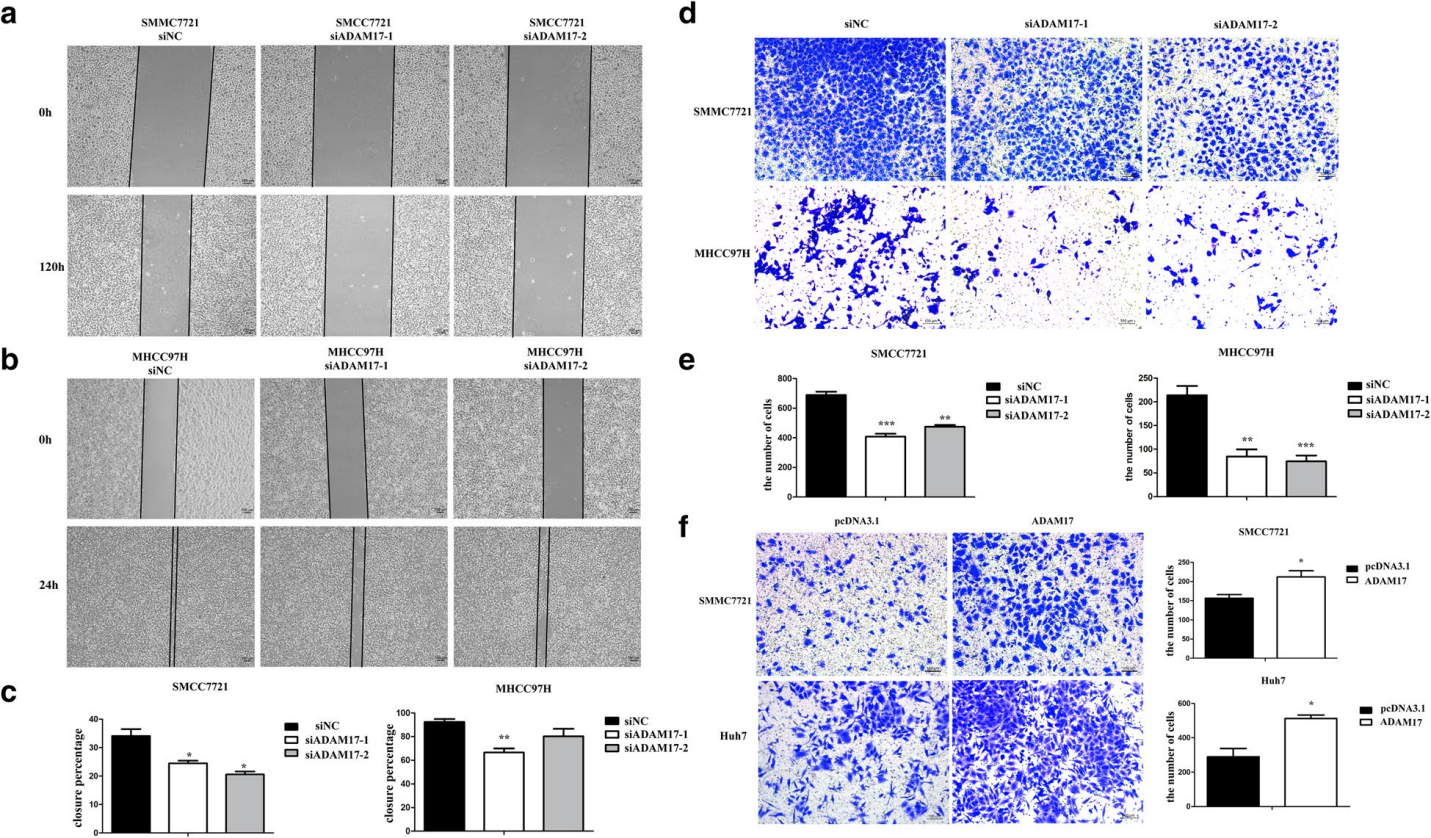
ADAM17 promotes the invasion and migration of hepatocellular carcinoma cells. **a**, **b** Effects of ADAM17 suppression on cell migration measured by the wound healing assay in SMMC7721 and MHCC97H cells (magnification, × 100). **c** Quantification of cell closure percentage. **d** Knockdown of ADAM17 impairs the invasive capacities of SMMC7721 and MHCC97H cells. **e** Quantification of cell invasion through Matrigel with each cell treatment (***P < 0.001 based on the Mann–Whitney U test). **f** Overexpression ADAM17 up-regulate invasion ability in Huh7 and SMMC7721 cells (magnification, × 100)

To assess the role of ADAM17 in the invasion and migration of hepatocellular carcinoma. We silenced ADAM17 in HCC cells (MHCC97H, SMMC7721) using small interfering RNAs (siRNAs). We found that silencing ADAM17 obviously changed the cell morphology (Additional file 1: Figure S1). To determine whether ADAM17 is associated with cell motility and metastasis, we performed invasion and wound healing assays. The number of migrated MHCC97H and SMMC7721 cells was significantly decreased following ADAM17 silencing (P < 0.05; Fig. 2d, e). Furthermore, in the wound healing assay (P < 0.05; Fig. 2a, b, c), we confirmed that the ranges of closure percentage were reduced by ADAM17 downregulation in MHCC97H and SMMC7721 cells. In addition we overexpression ADAM17 in Huh7 and SMMC7721, the invasion ability was obviously up-regulated (P < 0.05; Fig. 2f). Our results confirm that ADAM17 plays a pivotal role in hepatocellular carcinoma invasion and migration.

### The expression of MMP21 in HCC is associated with ADAM17

**Fig. 3:**
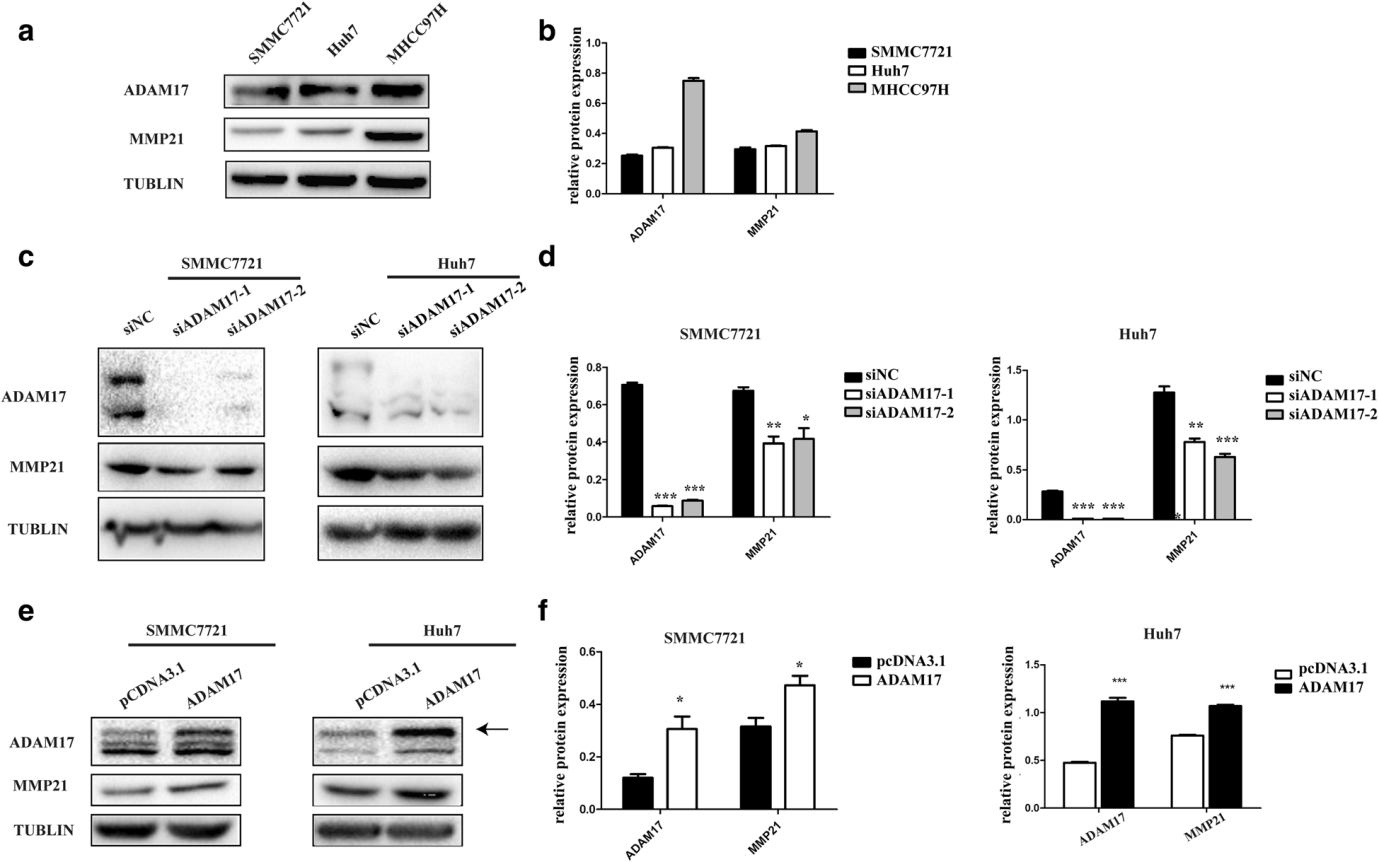
Knockdown and overexpression of ADAM17 in HCC cells affect MMP21 expression. **a**, **b** Expression of MMP21 in SMMC7721, Huh7 and MHCC97H cells. **c**, **d** The expression of MMP21 in SMMC7721 and Huh7 cells treated with ADAM17 siRNA. **e**, **f** The expression of MMP21 in SMMC7721 and Huh7 cells overexpressed ADAM17. (***P < 0.001, **P < 0.01, *P < 0.05)

Studies have shown that MHCC97H has the highest metastatic potential compared to other hepatocellular carcinoma cells. We examined MMP21 expression in three kinds of hepatocellular carcinoma cells by western blot (Fig. 3a, b). Our data indicate that the expression of MMP21 is significantly higher in MHCC97H cells than in Huh7 and SMMC7721 cells, which have lower metastatic potential, suggesting that MMP21 may be associated with tumor cell metastasis. Immunohistochemistry analysis showed a positive correlation between ADAM17 and MMP21 in patients with liver cancer (Table 2). To determine whether ADAM17 has an effect on MMP21 expression and activity, we first used siRNA to knockdown ADAM17 in Huh7 and SMMC7721 cells. As shown in Fig. 3c, d, we observed that the expression of MMP21 was significantly downregulated in the siRNA group (Huh7 and SMMC7721) in comparison with expression in the control. In addition, we also overexpressed ADAM17 in Huh7 and SMMC7721 cells, and the expression of MMP21 was markedly increased in the ADAM17 overexpression group compared with that in the control (Fig. 3e, f). This demonstrated that ADAM17 can regulate the expression of MMP21.

### ADAM17 influence HCC invasion and migration through MMP21

**Fig. 4:**
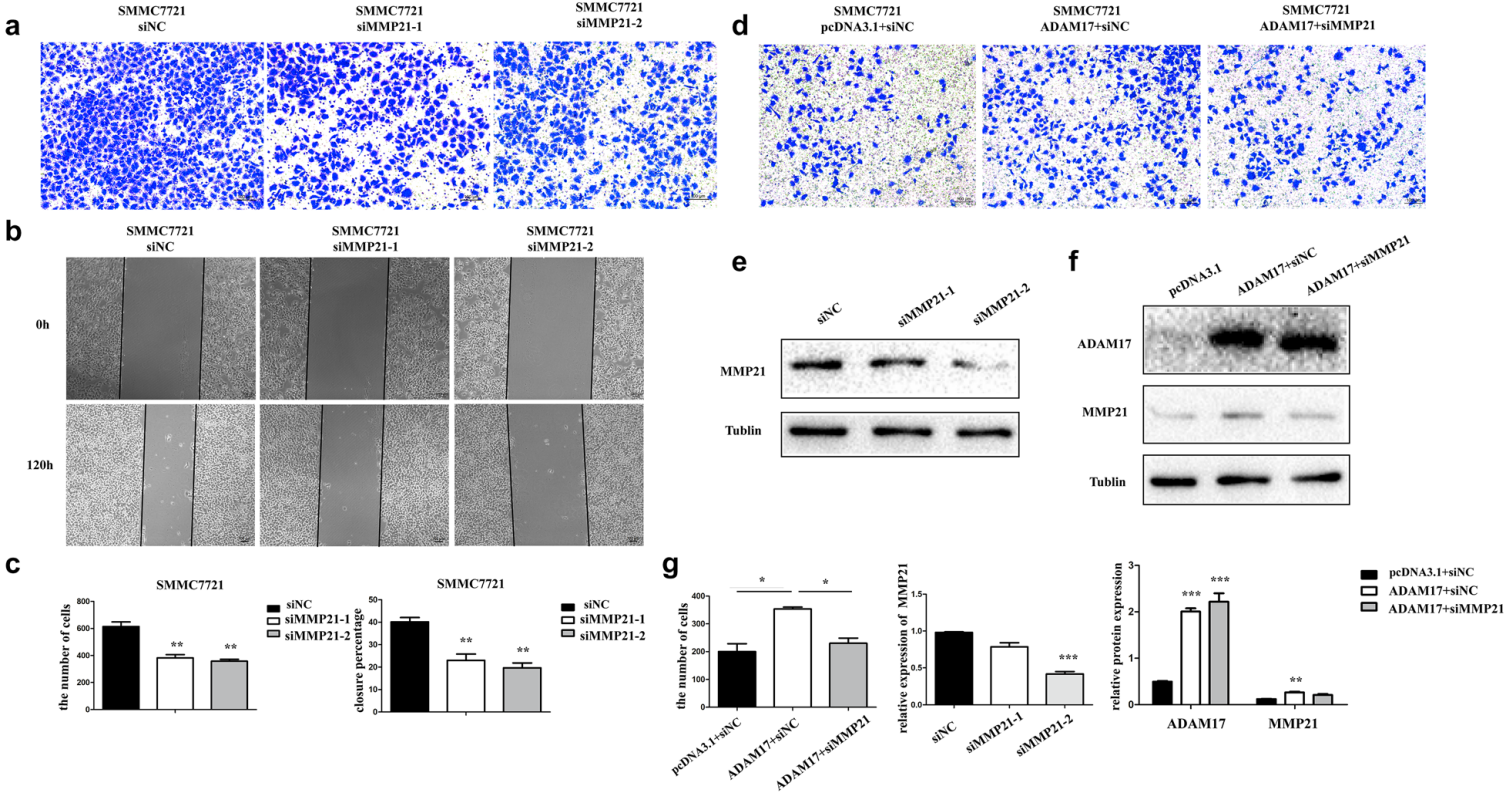
The expression of MMP21 influence HCC cell invasion and migration. **a**, **c** Knockdown of MMP21 decrease the invasion ability of SMMC7721. **b**, **c** Effects of MMP21 suppression on cell migration measured by the wound healing assay in SMMC7721 **d**, **g** ADAM17 regulate invasion ability through MMP21 in SMMC7721. **e**, **g** siRNA knockdown the expression of MMP21 in SMMC7721. **f**, **g** ADAM17 regulated MMP21 expression in SMMC7721. (***P < 0.001, **P < 0.01, *P < 0.05)

To further clarify that ADAM17 affects the invasion and migration of HCC through MMP21. We knockdown MMP21 with siRNA-MMP21, and found that the invasion ability of HCC was obvious down-regulated (Fig. 4a). Furthermore, in the wound healing assay the closure percentage was reduced when MMP21 was knockdown (Fig. 4b). The data was statistically significant (Fig. 4c). In addition, we overexpressed ADAM17 while knockdown MMP21 and found that the invasion ability was up-regulated when ADAM17 was overexpressed, while when we knockdown MMP21, the up-regulation was disappears (Fig. 4d, f, g). Figure 4e, f, g displays the overexpression and knockdown effects.

### ADAM17 expression influences mMMP21 secretion

**Fig. 5:**
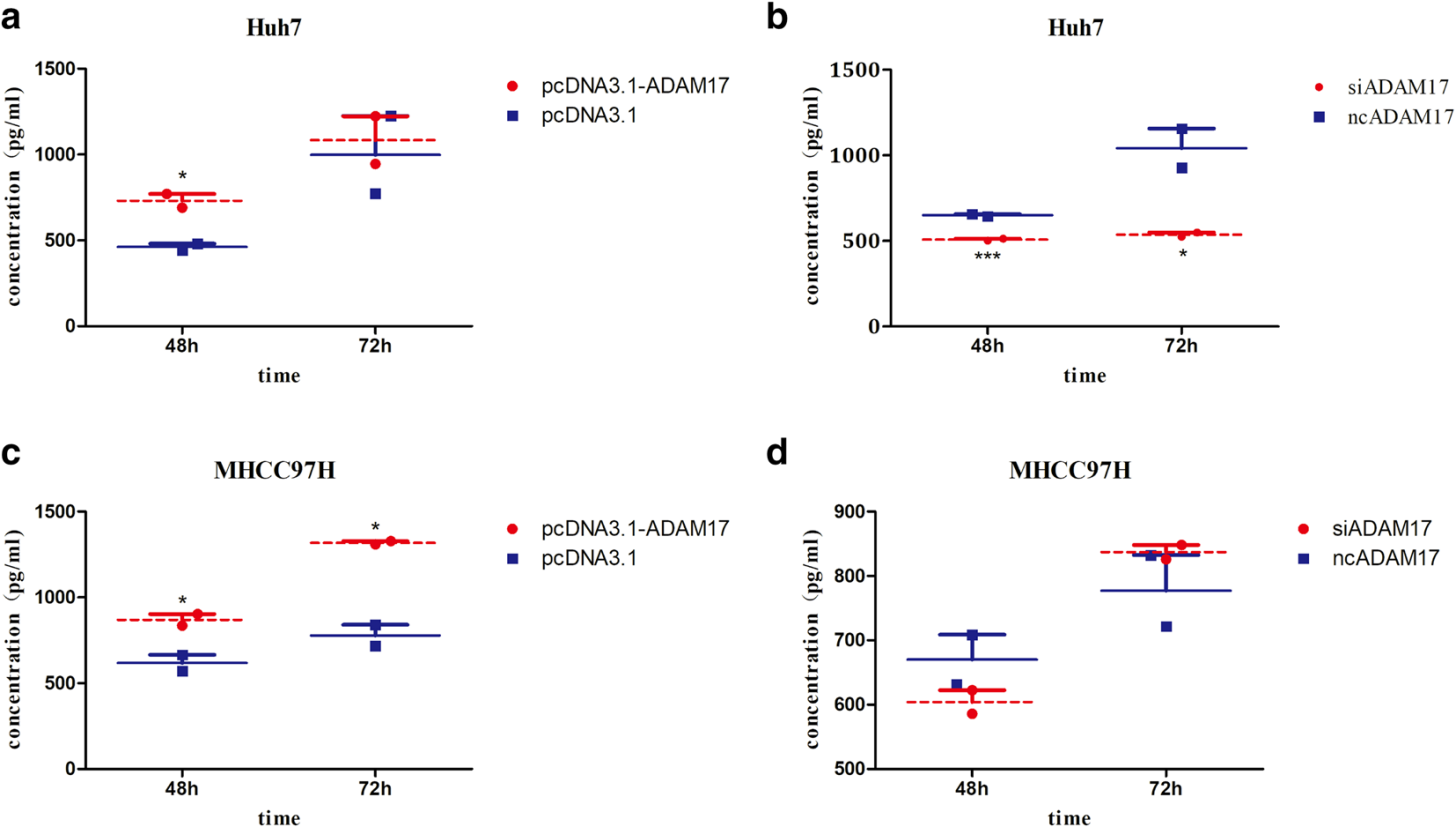
The secretion level of MMP21 in HCC cells. **a** Overexpression of ADAM17 enhanced the MMP21 secretion level in Huh7 cells. **b** Knockdown of ADAM17 reduced MMP21 secretion in Huh7 cells. **c** Overexpression of ADAM17 enhanced the MMP21 secretion level in MHCC97H cells. **d** Knockdown of ADAM17 reduced MMP21 secretion in MHCC97H cells (***P < 0.001, **P < 0.01, *P < 0.05)

Next, we investigated MMP21 secretion by ELISA. In Huh7 cells, supernatant from ADAM17 overexpression (pcDNA3.1(+)-ADAM17) or control (pcDNA3.1(+) conditions was harvested for measurements of MMP21 secretion over a period of 48 and 72 h after transfection without changing the media. We found that MMP21 secretion increased in pcDNA3.1(+)-ADAM17 transfectants at both time points (48 h and 72 h) (Fig. 5a), and the secretion ratio increased significantly compared with the control at 48 h, but there was no difference at 72 h. Furthermore, MMP21 secretion with siRNA-ADAM17 or control transfection in Huh7 cells was detected. The secretion difference between the two groups was remarkable at 48 h and 72 h (Fig. 5b). To further verify the results, we also tested MMP21 secretion levels in MHCC97H cells. The overexpression of ADAM17 enhanced the MMP21 secretion level at both 48 h and 72 h (P < 0.05, Fig. 5c). However, there was no significant difference in MMP21 secretion in MHCC97H cells when ADAM17 was knocked down (Fig. 5d). These data confirmed that ADAM17 influences mMMP21 secretion, but not just ADAM17 can influence the secretion of mMMP21.

### ADAM17 and MMP21 interact with each other in Huh7 cells, and the prodomain of MMP21 may be cleaved by ADAM17

**Fig. 6:**
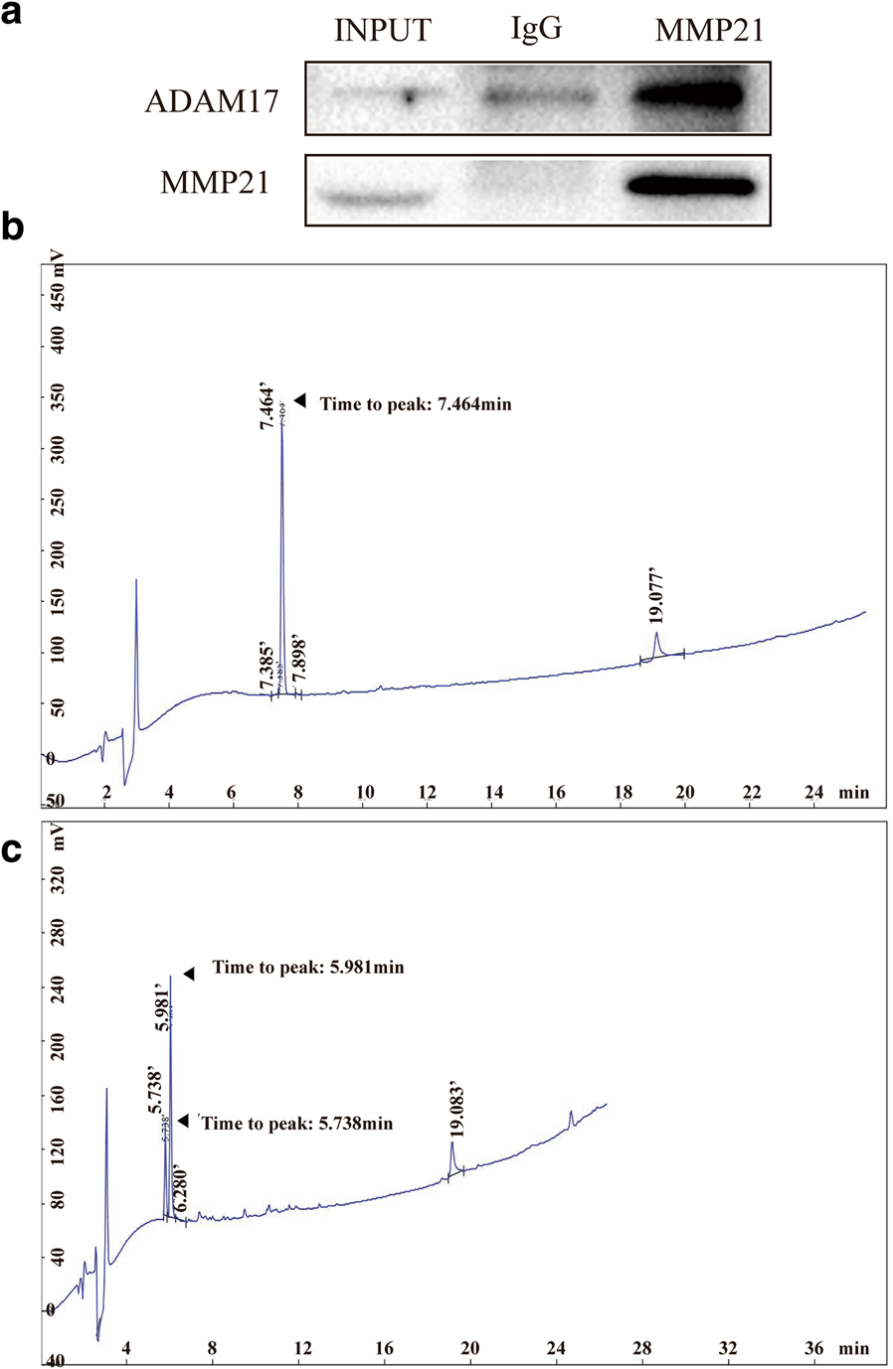
ADAM17 and MMP21 interact with each other in Huh7 cells, and the prodomain of MMP21 may be cleaved by ADAM17. **a** Immunoprecipitation analysis of the association between ADAM17 and MMP21 in Huh7 cells. **b** Analysis of a mixture of pro-MMP21 peptide with reaction buffer by UPLC-MS. (C) Analysis of a mixture of pro-MMP21 peptide with recombinant adam17 by UPLC-MS

The IP approach was applied to further prove the correlation between MMP21 and ADAM17. As shown in Fig. 6a, ADAM17 and MMP21 exhibited cross-linking in Huh7 cells. TNFα, a novel substrate for ADAM17 can be cleaved at a special site, Pro-Leu-Ala-Gln-Ala-|-Val-Arg-Ser- Ser-Se. Interestingly, the predomain of MMP21 contains a similar amino acid sequence, Leu-Ala-Gln-Ala-Val-Arg. Thus, we prepared a peptide (ALAEAVRRFQ) representing the sequence of pro-MMP21 and recombinant adam17 (rhTACE). The synthesized peptide was incubated with purified protease rhTACE, and then products were analyzed by reverse phase HPLC. As the data show, two main peaks were observed in the experimental group, while there was only one main peak in the control group (Fig. 6b, c), and the enzyme cleaved the peptide. Taken together, the results confirmed that ADAM17 participated in the maturation of MMP21.

## Discussion

HCC is one of the most prevalent human cancers. Tumor invasion is the main cause of mortality in patients with HCC [16–19]. Recent studies have determined that ADAM17 dysfunction may play an important role in tumor invasion [20]. In 2016, CD133-expressing CSCs were shown to be responsible for cell invasion and migration after radiation, and their radiation-induced metastatic potential could be prevented by suppression of ADAM17 [21]. A recent study proved that ADAM17 promotes cell invasion and migration through the integrinβ1 pathway in HCC [22]. In our study, we first used a database to confirm that ADAM17 was correlated with the OS and PFS of patients with HCC (Additional file 2: Figure S3). Later, we further identified that ADAM17 was markedly increased in HCC tissue samples. Moreover, we found that downregulation of ADAM17 could significantly suppress the invasion of MHCC97H and SMCC7721 cells. Therefore, ADAM17 may represent a novel target in the progression of HCC.

ADAM17 was originally identified as an enzyme responsible for processing TNFα from a precursor to a soluble circulating form [3, 23]. As a protein cleaved by ADAM17, TNFα seems to lack a definable consensus cleavage motif [19]. However, statistics in the MEROPS database showed that there is orderliness. It is more selective for alanine at the P1 position and revealed a preference for valine at the P1′ position among 60 cleavage sites registered in this database. Recently, some studies demonstrated that cleavage site specificities were in excellent agreement with the information derived from the MEROPS database [24, 25]. Interestingly, compared with the cleavage peptide (LAQAVRSS), we found that the prodomain of MMP21 has a similar peptide sequence (LAQAVR) through the Swiss Prot database. Furthermore, MMP21 was recently shown to play an important role in tumor processing [14–16, 26]. In our study, MMP21 was found to be upregulated in hepatocellular carcinoma and associated with microvascular invasion. In addition, the high expression of MMP21 in MHCC97H cells is often accompanied by high metastatic potential. Importantly, downregulation of ADAM17 could decrease the expression of MMP21, while overexpression of ADAM17 can markedly increase the expression of MMP21. Furthermore, ADAM17 can positively regulate the secretion of MMP21. Next, we confirmed that ADAM17 coimmunoprecipitated with MMP21 in the Huh7 cell line. On the other hand, it was further indicated by high-performance liquid chromatography that the precursor of MMP21 may be cleaved by ADAM17 protease.

## Conclusion

Our study demonstrated that increased ADAM17 expression may have contributed to HCC metastasis and progression. We found that ADAM17 and MMP21 expression in HCC was significantly higher than that in normal liver tissues and was associated with microvascular invasion both in vivo and in vitro. We first showed that ADAM17 was positively correlated with MMP21 in HCC. Overexpression of ADAM17 may improve tumor processing by cleaving the prodomain of MMP21 and activating it. In our study, we further expose the new mechanism of ADAM17 in HCC metastasis. Thus, ADAM17 is likely to be an attractive target for inhibiting HCC metastasis.

## Supplementary information

**Additional file 1: Figure S1.** The cell morphology was changed when silencing ADAM17 in MHCC97H.

**Additional file 2: Figure S2.** **a**. High expression of ADAM17 correlated with short OS(p = 0.037), **b**. High expression of ADAM17 correlated with short PFS(p = 0.04).

## Data Availability

The data and materials used to support the findings of this study are available from the corresponding author upon request.
