# Supplementary figures and images for: ADAM17 promotes the invasion of hepatocellular carcinoma via upregulation MMP21
Source: Cancer Cell Int. 2020 Oct 21;20:516. doi: 10.1186/s12935-020-01556-6 (PMC7579888; doi:10.1186/s12935-020-01556-6)

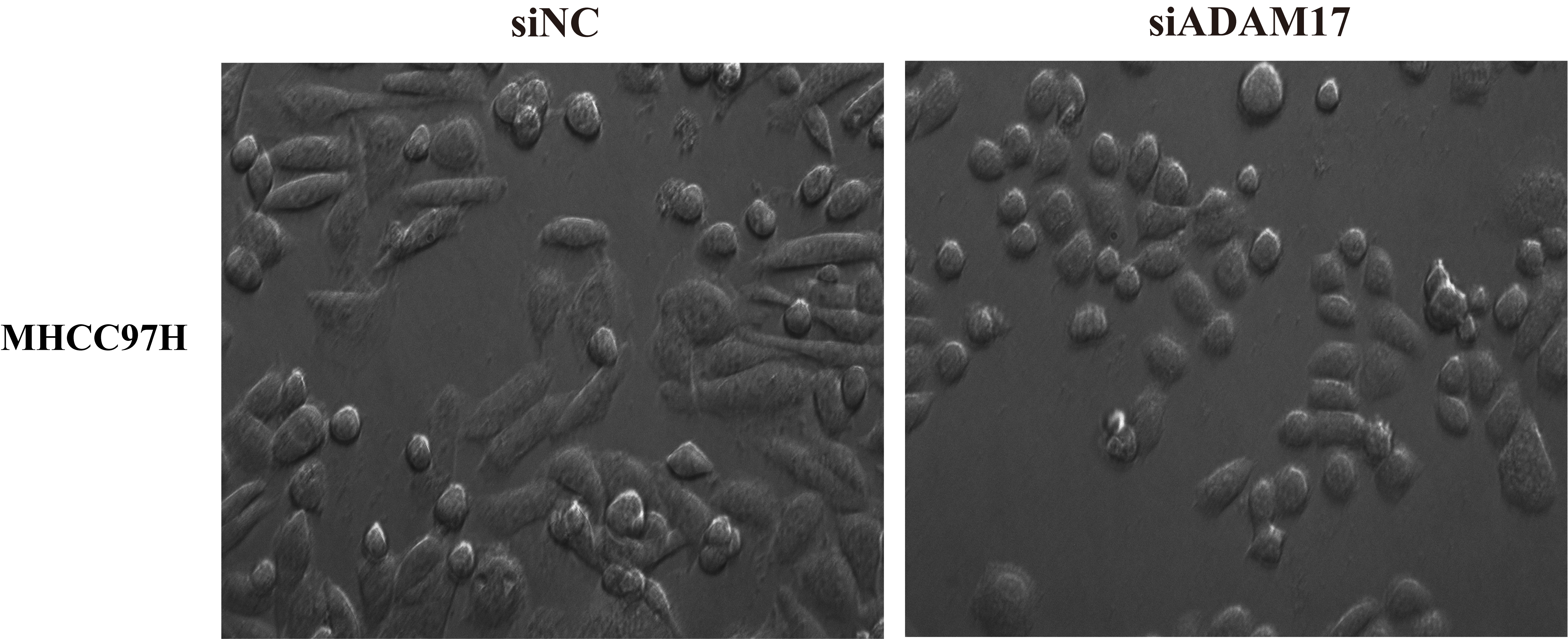

Supplement: Supplementary file 1 — Additional file 1: Figure S1. The cell morphology was changed when silencing ADAM17 in MHCC97H. [file 12935_2020_1556_MOESM1_ESM.tif]

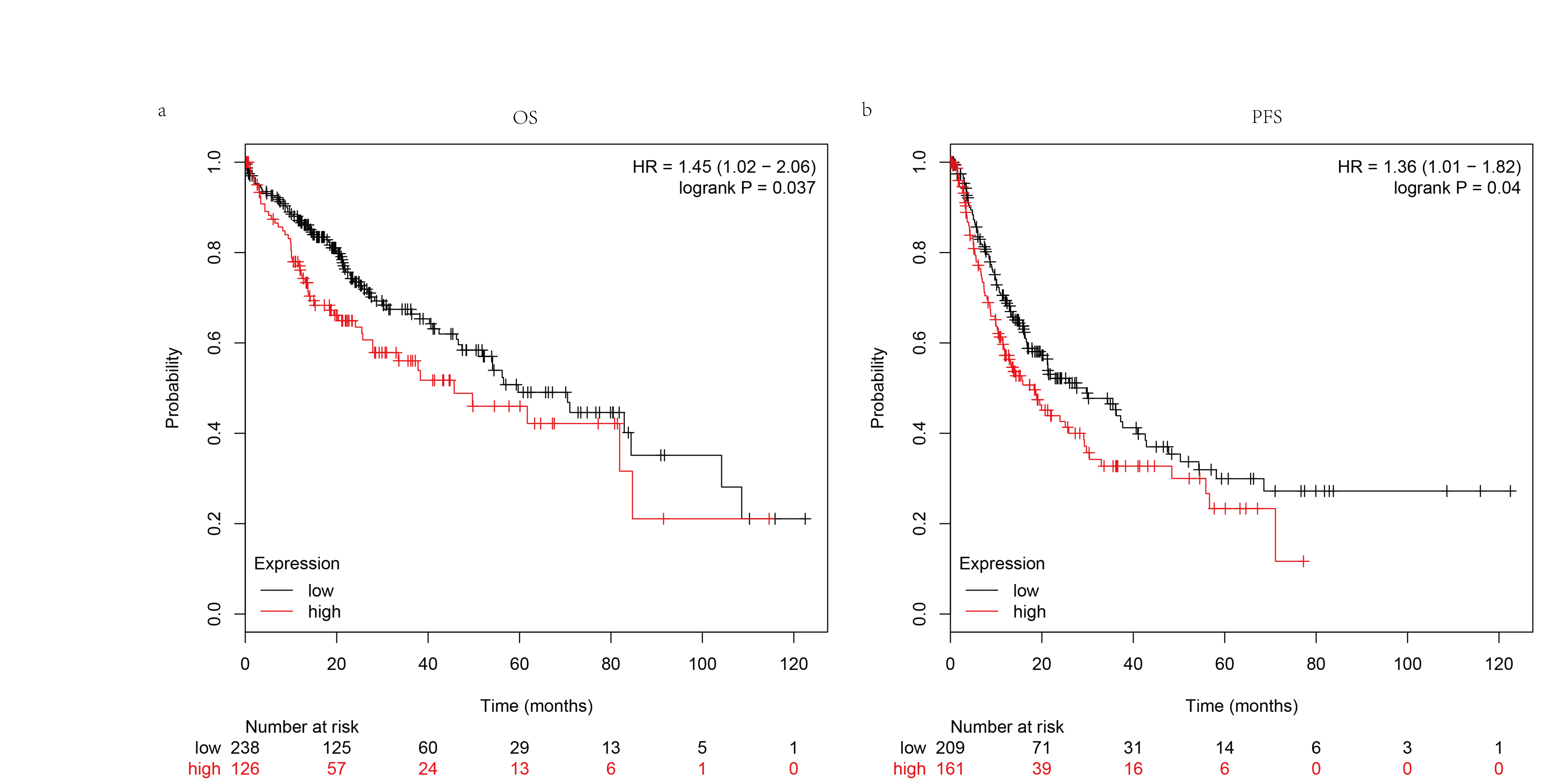

Supplement: Supplementary file 2 — Additional file 2: Figure S2. a. High expression of ADAM17 correlated with short OS(p = 0.037), b. High expression of ADAM17 correlated with short PFS(p = 0.04). [file 12935_2020_1556_MOESM2_ESM.tif]
